# Bibliometric analysis and visualization mapping of herpes zoster vaccine publications from 1999 to 2024

**DOI:** 10.3389/fmed.2025.1516450

**Published:** 2025-07-10

**Authors:** Wenli Gao, Dongyang Gao, Lin Li, Juan Li, Xinyi Yao, Junling Zhang

**Affiliations:** ^1^Graduate School, Tianjin University of Traditional Chinese Medicine, Tianjin, China; ^2^Beijing University of Chinese Medicine, Beijing, China; ^3^Dermatology Department, Tianjin Academy of Traditional Chinese Medicine Affiliated Hospital, Tianjin, China

**Keywords:** herpes zoster, Zostavax, Shingrix, CiteSpace, VOSviewer

## Abstract

**Background:**

Herpes Zoster (HZ) is a viral skin disease caused by reactivation of latent Varicella Zoster Virus (VZV) in human ganglia, presenting with unilateral neuropathic pain and vesicular rash. HZ imposes significant burden on patients and healthcare systems, often complicated by postherpetic neuralgia (PHN). The live attenuated zoster vaccine (ZVL, Zostavax) reduces HZ risk by 51% and PHN by 65% in adults ≥60 years. The recombinant zoster vaccine (RZV, Shingrix) shows approximately 90% efficacy in adults ≥50 years. Despite withdrawal of Zostavax from the U.S. market, RZV uptake remains suboptimal due to vaccine hesitancy and provider knowledge gaps. This study aims to analyze global research trends on HZ vaccines using bibliometric methods.

**Methods:**

Publications on “herpes zoster” and “zoster vaccine” were retrieved from the Web of Science Core Collection (WoSCC) database (1999–2024). Bibliometric and visualization tools including CiteSpace, VOSviewer, and R-bibliometrix were applied to analyze contributions by countries, institutions, journals, authors, references, and keywords.

**Results:**

A total of 719 articles from 261 journals across 56 countries were included. The United States led publications, with GlaxoSmithKline (GSK) as the most prolific institution. *Vaccine* was the most prolific journal; Levin et al. was the most productive author. The most cited article, “Efficacy of the Herpes Zoster Subunit Vaccine in Adults Aged 70 Years or Older,” by Cunningham AL, appeared in the *New England Journal of Medicine*. Frequently used keywords included “herpes zoster,” “vaccination,” “postherpetic neuralgia,” “efficacy,” “subunit vaccine,” and “safety”.

**Conclusion:**

This bibliometric study comprehensively summarizes HZ vaccine research over 25 years, offering insights to guide future research and clinical practice.

## 1 Introduction

Varicella Zoster Virus (VZV), a neurotropic *α*-herpesvirus, causes two clinically distinct conditions: varicella (chickenpox) as primary infection, and herpes zoster (HZ) upon reactivation of latent virus residing in dorsal root or cranial nerve ganglia. Children who are susceptible to VZV develop chickenpox upon infection, and following recovery, the virus persists for life in a latent form within the dorsal root ganglia or cranial nerve ganglia. Reactivation is triggered by factors such as immunosenescence or immunodeficiency, resulting in HZ. The reactivation of latent VZV can occur when risk factors such as aging or cellular immunodeficiency arise, leading to the development of herpes zoster.

The typical disease duration is 2–3 weeks, extending to 3–4 weeks in elderly patients. Complications affect >10% of patients and include vision loss, neuropathic pain, and cerebrovascular events (1).

Global incidence ranges from 3 to 5 cases per 1,000 person-years (2) in the general population but increases sharply in immunocompromised groups, such as those with hematologic malignancies (up to 31/1,000 person-years) (3) or human immunodeficiency virus (HIV) infection (29.4–51.5/1,000 person-years), with recurrence rates between 13 and 26% (4). Postherpetic neuralgia (PHN), a chronic and often recurrent complication, particularly burdens elderly patients by prolonging pain and reducing quality of life.

Vaccination remains the cornerstone of HZ prevention. Two vaccines are currently licensed worldwide: the live attenuated zoster vaccine (ZVL, Zostavax) and the recombinant zoster vaccine (RZV, Shingrix). A notable ZVL is Zostavax, which has a vaccine efficacy (VE) of 51% against HZ and 65% against postherpetic neuralgia; however, VE significantly declines with the recipient’s age. RZV shows >90% VE (5), recommended for healthy adults ≥50 years and immunocompromised individuals ≥19 years (6). It contains recombinant VZV glycoprotein E (gE) combined with the AS01B adjuvant, inducing robust antigen-specific CD4^+^ T-cell and humoral responses. This vaccine employs a recombinant subunit of VZV glycoprotein E (gE) combined with the novel AS01B adjuvant, which elicits a stronger specific CD4 + T cell response and humoral immune response.

Despite proven efficacy and safety, HZ vaccine coverage lags behind other adult vaccines such as influenza and pneumococcal vaccines. Barriers include limited awareness and knowledge among healthcare providers and patients.

While this bibliometric study does not directly address vaccine uptake, it identifies research trends and emerging gaps in scientific communication, safety perceptions, and public awareness, which may indirectly inform policymaking and education strategies.

The past 25 years (1999–2024) represent a critical period capturing major milestones in HZ vaccine research, including the development, approval, and widespread implementation of both ZVL and RZV. This timeframe thus provides a comprehensive overview of the scientific progress and evolving challenges in this field. Moreover, recent developments—such as emerging vaccine hesitancy and disparities in vaccine access—underscore the urgency and relevance of synthesizing current research trends through bibliometric analysis.

This 25-year timeframe captures two transformative milestones in HZ vaccine development—Zostavax in 2006 and Shingrix in 2017—and their respective impacts on global research priorities. Recent debates surrounding vaccine hesitancy and post-pandemic immunization dynamics further underscore the timeliness and necessity of this bibliometric assessment.

Bibliometric analysis is a quantitative approach for mapping research output, trends, and hotspots, which can identify knowledge gaps and emerging research fronts. The Web of Science Core Collection (WoSCC) serves as a principal database for such analyses (7), with analytical tools like CiteSpace (8, 9), VOSviewer (10), and R-bibliometrix widely adopted. While bibliometric studies on general HZ literature exist, focused systematic bibliometric analyses specifically targeting HZ vaccine research remain scarce. Recent authoritative reviews have highlighted the importance and timeliness of bibliometric approaches to vaccine research to guide future scientific priorities and policy-making (11–13).

Accordingly, this systematic review employs bibliometric and visualization methods to comprehensively analyze the global research landscape of HZ vaccines over the past 25 years. Our findings aim to inform researchers, clinicians, and policymakers by highlighting research trends, gaps, and directions, ultimately supporting enhanced vaccine development, awareness, and uptake.

## 2 Materials and methods

### 2.1 Data sources and search strategies

**Figure 1:**
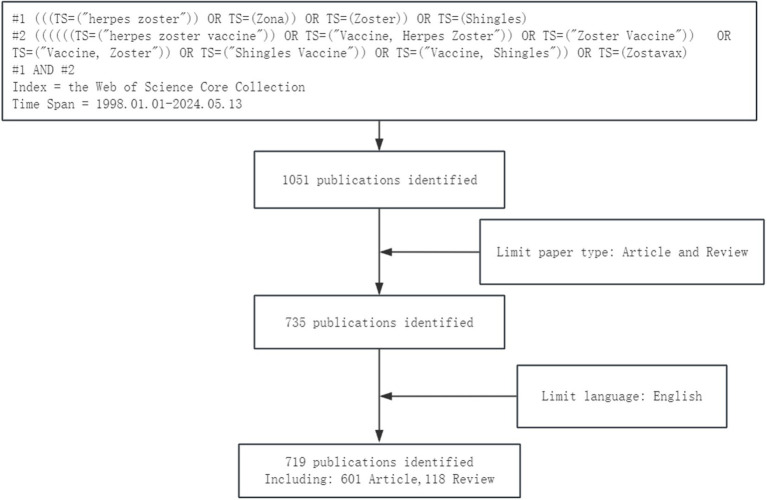
Flowchart of the literature search and selection process.

The search keywords were established using Medical Subject Headings (MeSH) and free terms. The search was conducted within the Web of Science Core Collection (WoSCC), specifically in the Science Citation Index Expanded (SCI-EXPANDED). The search strategy consisted of: #1: (((TS = (“herpes zoster”)) OR TS = (Zona)) OR TS = (Zoster)) OR TS = (Shingles)), and #2: (((((TS = (“herpes zoster vaccine”)) OR TS = (“Vaccine, Herpes Zoster”)) OR TS = (“Zoster Vaccine”)) OR TS = (“Vaccine, Zoster”)) OR TS = (“Shingles Vaccine”)) OR TS = (“Vaccine, Shingles”)) OR TS = (Zostavax). The final result was obtained by combining #1 AND #2. The search was performed on May 13, 2024, with the time frame set from January 1, 1999, to the present. Article types were limited to Articles or Reviews, and the language was restricted to English. Ultimately, 719 documents were included, consisting of 601 Articles and 118 Reviews. The search results were exported in “Plain Text Format” and “EndNote Desktop” format, including “full record with references cited” for bibliometric analysis. All downloaded data were independently retrieved by two researchers (Wenli Gao and Dongyang Gao). A flowchart of the literature search process is presented in Figure 1.

Unlike traditional systematic reviews, which qualitatively synthesize study outcomes and methodologies, bibliometric analyses adopt algorithm-based, quantitative approaches with visual mapping. In this study, human validation was applied during keyword merging, cluster interpretation, and software output refinement to ensure accuracy and relevance.

### 2.2 Data extraction and analysis

The text files were imported into CiteSpace 6.3. R1 Advanced, VOSviewer 1.6.18, Bibliometrix 4.2.4, the online analysis platform,^1^ and Scimago Graphic. Using these specialized software tools, bibliometric analyses were conducted on key information related to herpes zoster and herpes zoster vaccines obtained from the WOSCC database, and the results were visualized in graphical format.

CiteSpace, developed by Professor Chaomei Chen, is a bibliometric analysis software known for its robust visualization capabilities. In this study, it was used to analyze countries, dual-map overlays of journals, disciplines, references, and keyword co-citation bursts and clustering. VOSviewer, developed by the Centre for Science and Technology Studies at Leiden University, is a user-friendly bibliometric visualization tool with strong mapping abilities. In this study, it primarily served to analyze institutions, authors, and keywords. Bibliometrix is an R package that offers a straightforward and comprehensive set of analytical tools, enabling the visualization of inter-country relationships, publication timelines for journals and authors, as well as keyword clouds. Additionally, the online analysis platform (see text footnote 1) was utilized to analyze the annual cumulative publication volumes and collaboration relationships among countries. Scimago Graphic was used to provide a visual representation of publication output and collaboration intensity between nations.

## 3 Results

### 3.1 Annual growth trends in publications

**Figure 2:**
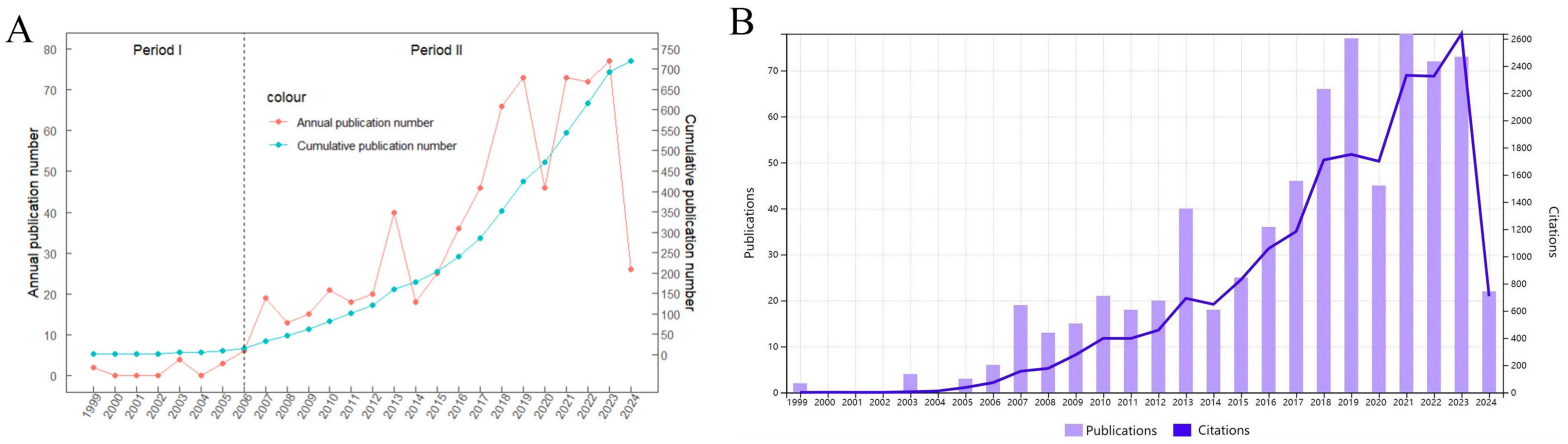
**(A)** Annual number and cumulative volume of herpes zoster vaccine-related publications from 1999 to 2024. **(B)** Annual publication volume with corresponding citation frequency.

From January 1, 1999, to May 13, 2024, a total of 719 publications related to herpes zoster and its vaccines were retrieved from the SCI-E database of WOSCC. Figure 2 presents a line graph showing the annual publication count and cumulative publication volume on the relevant topics, while Figure 2B displays a bar chart of the publications alongside a line graph of citation frequencies. Overall, the publication volume shows an upward trend, with some fluctuations in individual years, indicating increasing research attention in this area. The number of publications peaked in 2021, with 77 articles published and cited 2,331 times. As of May 13, 2024, there have been 26 publications this year, with an expectation of continued growth. The total number of citations for the included publications in this study is 19,563, with an average citation frequency of 27.21. The H-index for this academic field from 1999 to 2024 is 66.

### 3.2 Distribution of countries/regions

**Figure 3:**
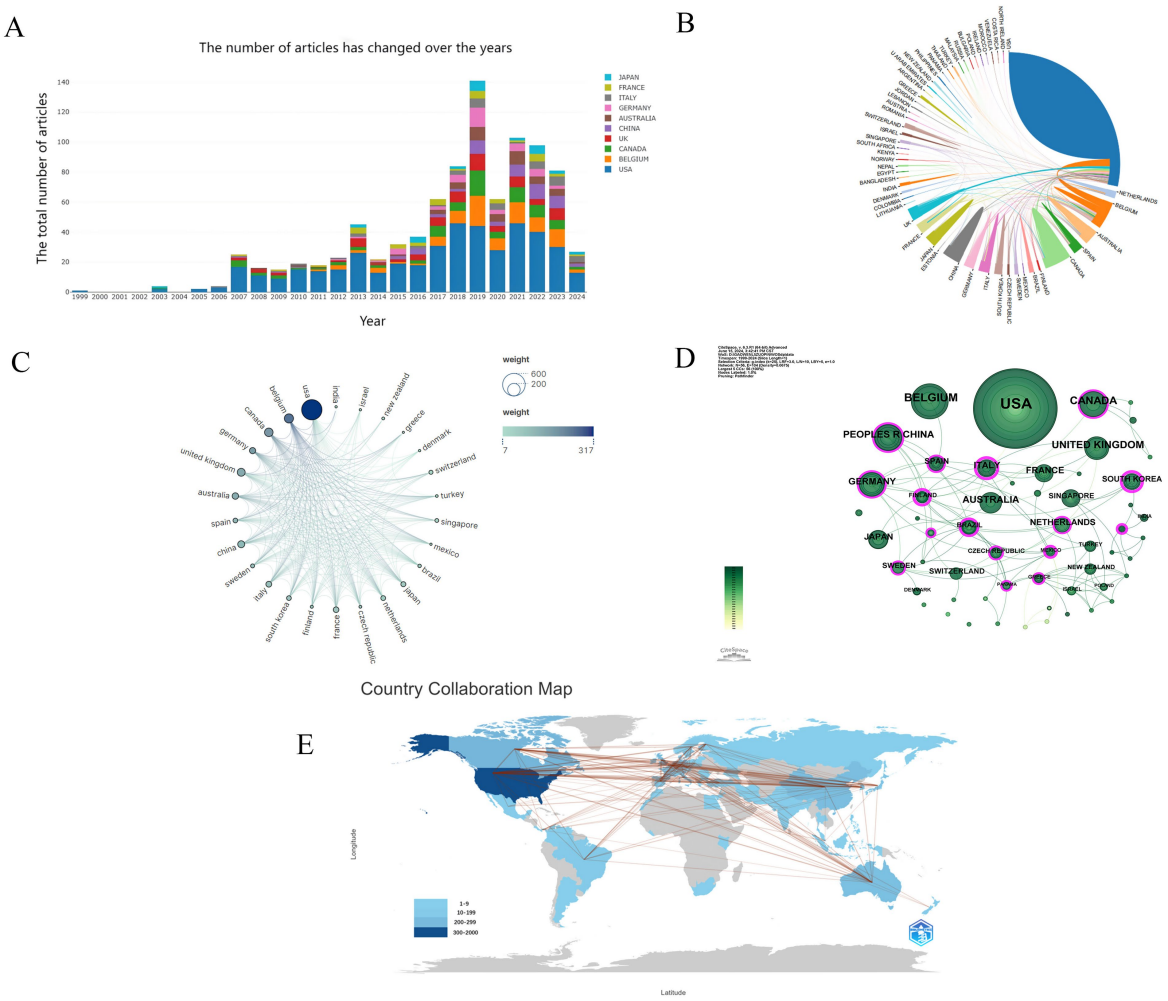
**(A)** Shows the annual publication trends of the top 10 countries from 1999 to 2024; **(B)** illustrates the proportion of the 10 most productive countries in clinical trials for herpes zoster vaccination over the past 25 years; **(C)** provides a visual map of international collaboration among countries and regions; **(D)** presents a network visualization of relationships between countries; **(E)** depicts a world map based on publication volume and inter-country connections.

In this study, we performed a comprehensive bibliometric visualization analysis of the herpes zoster (HZ) vaccine research landscape, incorporating 719 publications from 56 countries and regions. Figure 3A depicts the top ten countries ranked by publication volume from 1999 to 2024, along with their respective proportions. The United States leads markedly with 443 publications, accounting for 61.61% of the total output, yet demonstrates relatively low betweenness centrality, suggesting limited collaborative interactions with other nations. Belgium (91 publications, 12.66%) and Canada (79 publications, 10.99%) follow in publication counts. Notably, the development of the first live attenuated HZ vaccine, Zostavax, by Oxman et al. (2006) corresponds with a substantial increase in publication volume between 2006 and 2007. A subsequent minor peak occurred during 2012–2013, whereas publication output declined in 2019–2020, potentially attributable to the COVID-19 pandemic’s disruptive impact.

The top ten countries ranked by publication volume and their betweenness centrality scores—a network metric used in bibliometrics to measure a node’s influence within the collaboration network by assessing how often it lies on the shortest path between other nodes, thereby indicating its importance in facilitating connections—are summarized in Table 1. While the United States dominates in publication quantity, its centrality score is zero, indicating a relatively limited role in connecting other countries within the global research network. In contrast, Finland (centrality 0.52), Italy (0.42), and South Korea (0.41) exhibit notably higher centrality values, suggesting their pivotal roles as hubs facilitating multinational research collaborations. This distinction highlights that, although the U.S. leads in output, several smaller countries play crucial roles in bridging international partnerships and enhancing the cohesiveness of the herpes zoster vaccine research community. Betweenness centrality thus underscores how frequently a country lies on the shortest path between others, emphasizing its influence in knowledge dissemination and cooperative synergy (see Table 1).

**Table 1:** The top 10 countries ranked by publication volume and centrality.

Rank	Documents	Countries	Centrality	Centrality	Countries	Documents
1	443	USA	0	0.52	Finland	11
2	91	Belgium	0	0.42	Italy	37
3	79	Canada	0.2	0.41	South Korea	25
4	68	UK	0.07	0.37	Mexico	6
5	53	China	0.10	0.32	Nepal	3
6	46	Australia	0.07	0.27	Brazil	13
7	42	Germany	0.11	0.20	Canada	79
8	37	Italy	0.42	0.19	Greece	6
9	35	France	0.08	0.18	Panama	5
10	28	Japan	0	0.15	Spain	25

**Table 2:** The top 11 institutions with more than 15 publications.

Organization	Documents	Citations	Total link strength	Country
Glaxosmithkline	77	1,645	144	UK
Univ. Colorado	49	1,679	130	USA
CTR Dis Control & Prevent	47	1,229	60	USA
Merck & Co Inc.	41	1,422	72	USA
Duke Univ.	24	1,503	103	USA
Univ. Sydney	22	474	60	Australia
Durham Vet Med Ctr	17	1,296	77	UK
Dalhousie Univ.	17	865	66	Canada
Stanford Univ.	17	538	34	USA
Univ. Calif San Diego	16	1,203	74	USA
Univ. Alabama Birmingham	16	783	23	USA

Figure 3B further illustrates the distribution and collaborative dynamics among countries engaged in HZ vaccine clinical research. Node size represents publication output, node color encodes the average publication year, and edge thickness quantifies collaboration strength. Peripheral purple nodes denote countries with betweenness centrality exceeding 0.1, indicative of pivotal collaborative roles. Finland, Italy, and South Korea exhibit notably high centrality values of 0.52, 0.42, and 0.41, respectively, reflecting their prominent positions within international collaboration networks.

Figure 3C presents a visual network map of international cooperation, highlighting 26 countries with publication counts exceeding five. Node size correlates with publication volume, while edges represent inter-country collaboration ties. Figure 3D displays a country-level publication network generated by CiteSpace. Different node shapes correspond to distinct countries, with connecting lines representing cooperative linkages. Node size and coloration indicate publication quantity and temporal distribution, respectively; edge thickness reflects collaboration intensity. The presence of peripheral purple nodes further underscores countries with substantial betweenness centrality, signifying their integral roles in fostering global research partnerships.

Lastly, Figure 3E depicts a global map that integrates publication volume and inter-country connectivity, effectively illustrating the spatial distribution of research productivity and collaborative relationships. Color gradients correspond to publication volume, and connecting lines visualize cooperative interactions. Collectively, these bibliometric visualizations provide an insightful overview of the geographic distribution, research productivity, and collaborative networks characterizing the HZ vaccine research field.

### 3.3 Active authors and institutions analysis

**Figure 4:**
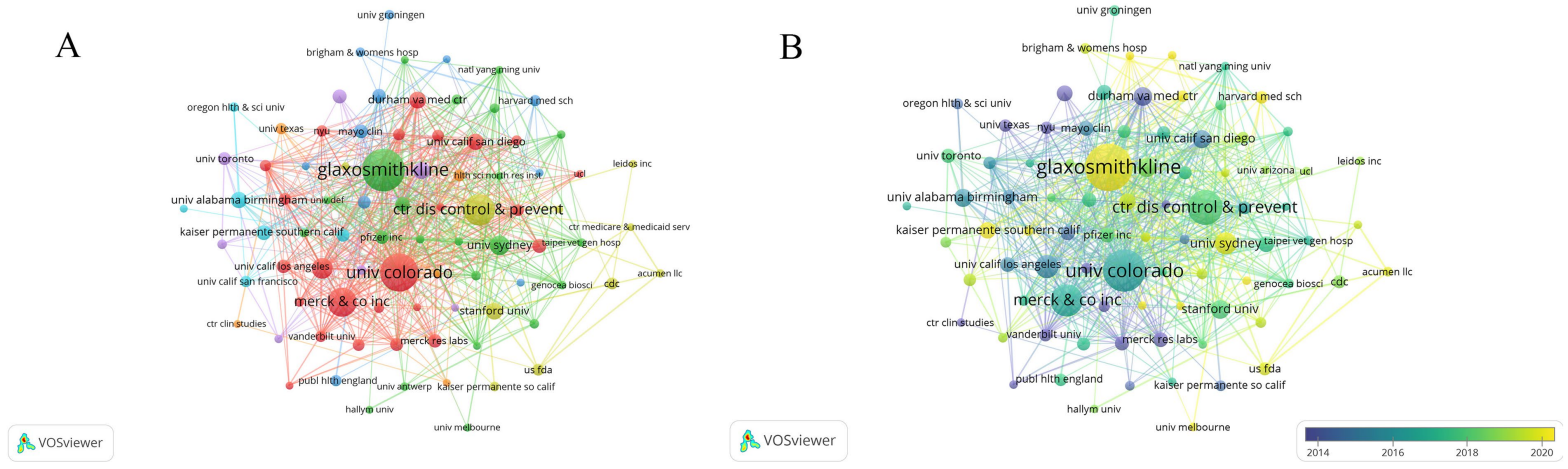
**(A)** Institutional collaboration network showing relationships among 88 institutions. Node size indicates number of publications; edge thickness denotes collaboration strength. **(B)** Timeline of institutional publications, with node color representing publication recency.

**Figure 5:**
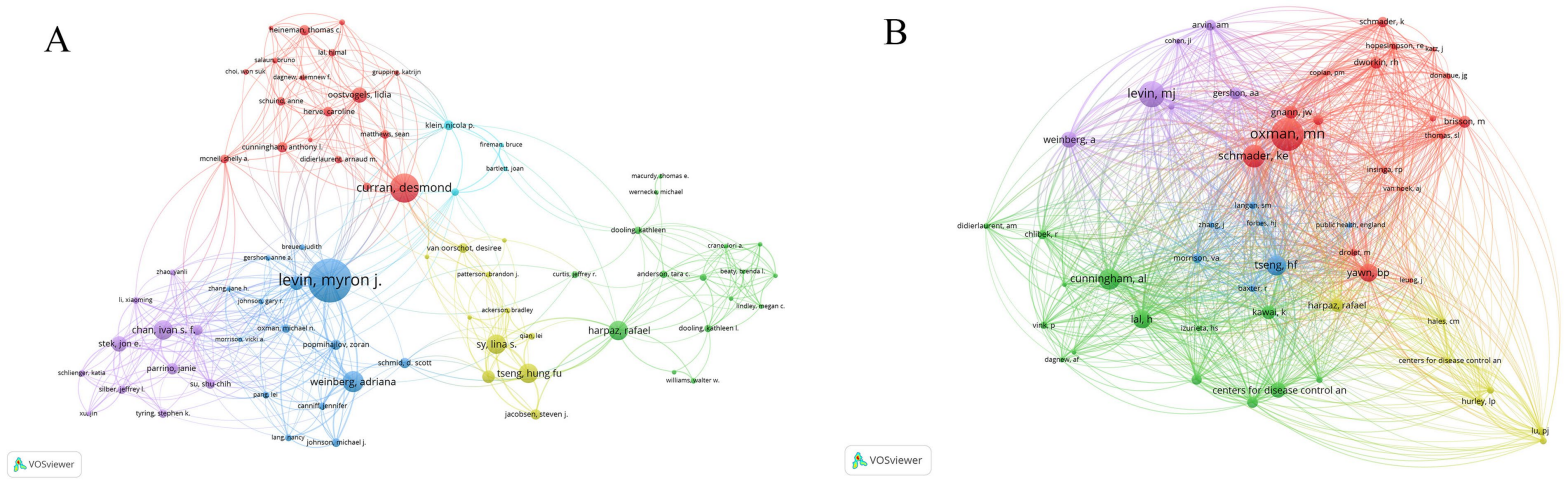
**(A)** Author collaboration network. Nodes represent authors with >5 publications; edge thickness indicates collaboration frequency. **(A)** Illustrates the author collaboration graph, showing a total of 82 authors with more than 5 publications; after removing those without connections, 75 authors remain. **(B)** Distribution of citation frequency among authors. Node size reflects total citations. **(B)** Indicates that there are 52 authors with a total of 50 citations.

**Figure 6:**
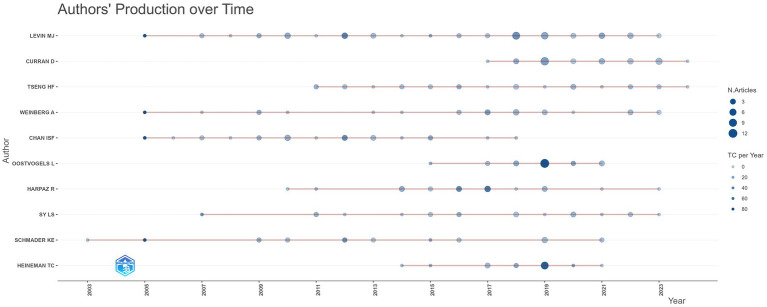
Author publication timeline with citation impact. Publication volume timeline of the top 20 authors in herpes zoster vaccine research. Point size reflects citation count per year.

In this study, we analyzed the collaboration and publication patterns of active institutions and authors involved in herpes zoster vaccine research. Among 1,215 institutions, 88 had published more than five articles, with 85 forming an interconnected collaboration network depicted in Figure 4. In this network, node size corresponds to the number of publications, node colors transition from cool to warm tones representing earlier to more recent publication years, and edge thickness indicates the strength of institutional collaborations. GlaxoSmithKline (GSK) in the UK leads with 77 publications, followed by the University of Colorado in the USA. Notably, seven of the top ten institutions are based in the USA, with the UK, Australia, and Canada each contributing one institution. Figure 4B further illustrates the temporal publication trends of these institutions, confirming that GSK and the University of Sydney not only have substantial output but also remain actively engaged in recent years. Table 2 lists the top 11 institutions with more than 15 publication. Regarding author contributions, a total of 3,398 authors participated in this field, with 82 authors having published more than five articles. After excluding unconnected authors, 75 remain in the collaboration network shown in Figure 5A. Levin et al. from the University of Colorado is the most prolific author with 45 publications, followed by Curran, Desmond from GSK (30 publications) and Weinberg, Adriana, also from the University of Colorado (22 publications). Despite these productive authors, the overall author collaboration network exhibits limited connectivity, suggesting room for enhancing cooperative efforts. Figure 5B highlights that 52 authors have accrued more than 50 citations, with Oxman ranking highest, reflecting his seminal influence in this research area. Figure 6 displays the publication timeline for the top 20 authors, with point sizes proportional to their annual citation counts; Levin et al. has maintained consistent high impact, while emerging contributors such as Curran et al. have demonstrated increasing productivity and citation influence.

Overall, these bibliometric visualizations, with clearly defined node sizes (publication counts), colors (publication year timelines), and edge thicknesses (collaboration strengths), provide an informative overview of the key contributors and collaboration patterns within the herpes zoster vaccine research community.

### 3.4 Journals and co-cited journals

**Figure 7:**
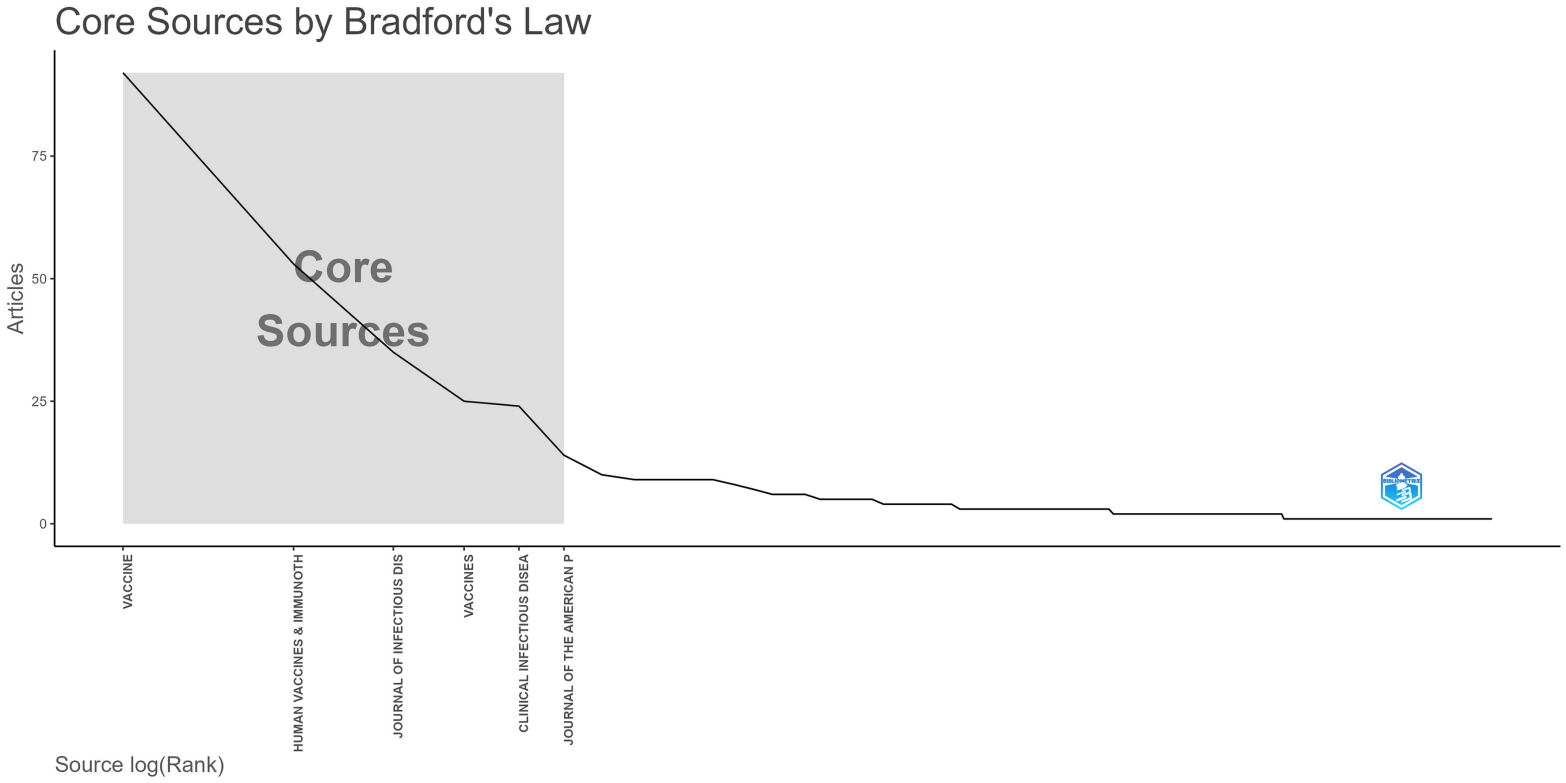
Core journals in herpes zoster vaccine research, based on Bradford’s Law. Node size indicates publication count; color reflects field classification.

**Figure 8:**
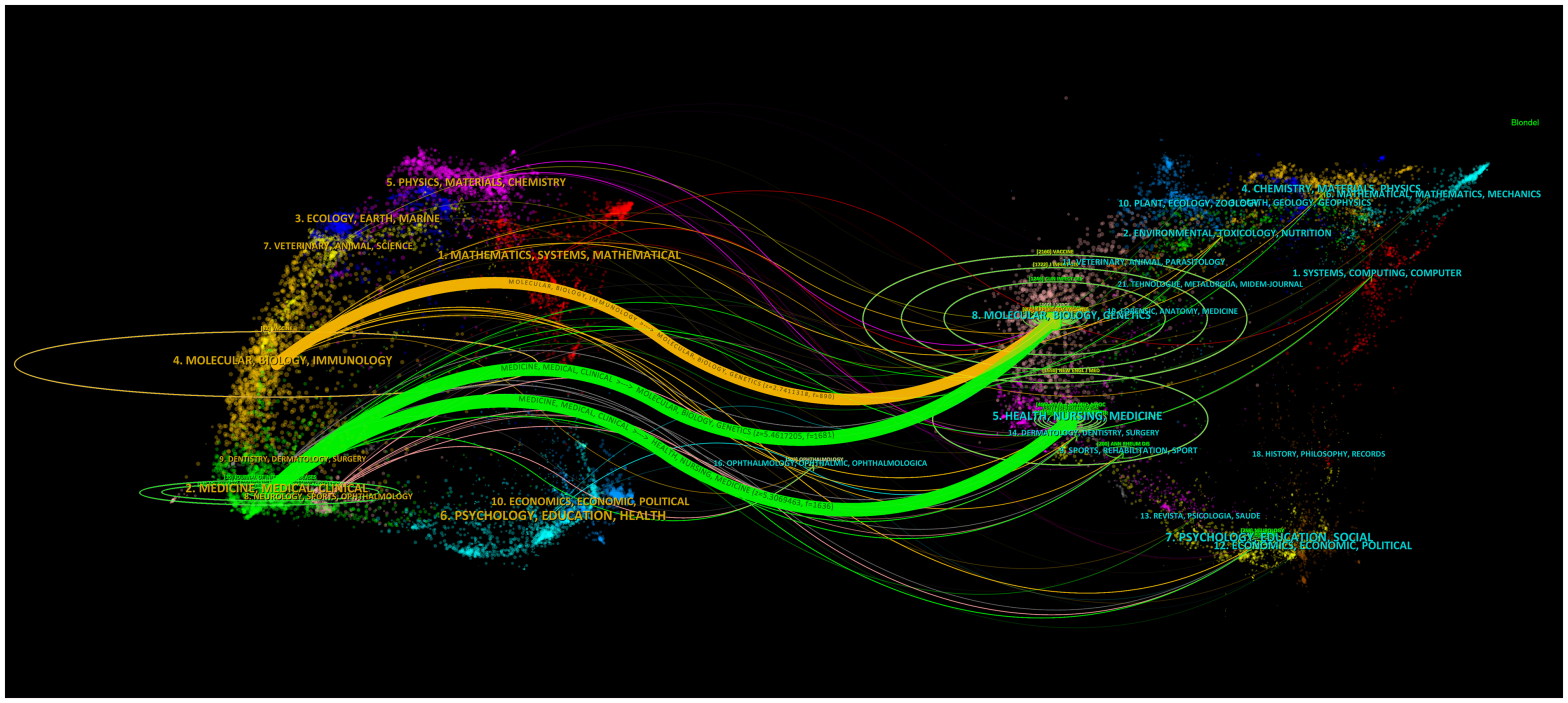
Dual-map overlay illustrating citation relationships between citing and cited journals. Line thickness indicates citation strength.

This study analyzed 261 journals, of which 21 published more than five articles related to herpes zoster vaccine research. According to Bradford’s Law, core journals in this field include *Vaccine*, *Human Vaccines & Immunotherapy*, *Journal of Infectious Diseases*, *Vaccines*, *Clinical Infectious Diseases*, and *Journal of the American Pharmacists Association*, primarily focusing on vaccines and infectious diseases (Figure 7). In Figure 7, node size represents the number of publications per journal, with larger nodes indicating higher publication volumes. Different colors correspond to various academic disciplines, allowing clear visualization of journal distribution across fields. To visualize citation patterns and disciplinary relationships, a journal dual-map overlay was utilized (Figure 8). This network displays citing journals on the left and cited journals on the right. Nodes’ sizes indicate publication volume within each journal category, while colors distinguish disciplinary areas. Connecting lines between nodes illustrate citation pathways, with line thickness and color intensity representing the frequency and strength of citation relationships. For example, the prominent orange path from molecular biology and immunology journals to molecular biology and genetics journals (z = 2.74, *f* = 890) highlights a strong citation linkage between these disciplines, demonstrating the cross-disciplinary flow of knowledge. Such visualization using node size, color, and edge thickness provides a comprehensive and intuitive understanding of the citation structure, improving interpretability for readers unfamiliar with complex bibliometric maps.

Table 3 presents the top ten cited and citing journals. The most cited journal is *Vaccine*, while the journal with the highest citation count is *New England Journal of Medicine*, with 566 citations, an impact factor of 158.5, and a JCR classification in the first quartile. This highlights its authoritative role and the high quality of publications referenced by herpes zoster vaccine research.

**Table 3:** The top ten citing and cited journals.

Rank	Journal	Counts	IF/JCR	Cited journal	Counts	IF/JCR
1	Vaccine	92	5.5/Q2	New Engl J Med	566	158.5/Q1
2	Hum Vacc Immunother	53	4.8/Q2	Vaccine	538	5.5/Q2
3	J Infect Dis	35	6.4/Q1	Clin Infect Dis	481	11.8/Q1
4	Vaccines	25	7.8/Q1	J Infect Dis	468	6.4/Q1
5	Clin Infect Dis	24	11.8/Q1	MMWR-Morbid Mortal W	340	33.9/Q1
6	J AM Pharm Assoc	14	2.1/Q4	JAMA-J Am Med Assoc	259	120.7/Q1
7	BMJ Open	10	2.9/Q2	Hum Vacc Immunother	237	4.8/Q2
8	AM J Prev Med	9	5.5/Q1	Ann Intern Med	237	39.2/Q1
9	BMC Infect Dis	9	3.7/Q3	Mayo Clin Proc	218	8.9/Q1
10	Expert Rev Vaccines	9	6.2/Q2	BMJ Open	160	2.9/Q2

### 3.5 Co-cited references and references burst

**Figure 9:**
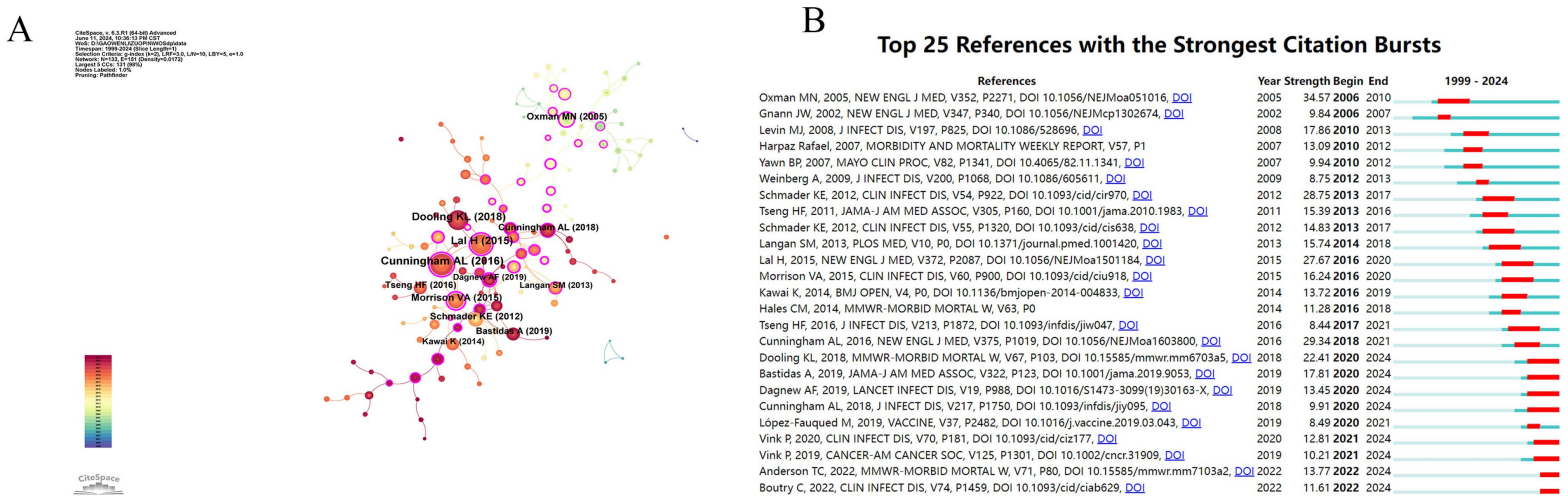
**(A)** Co-citation network of top references. Node size reflects citation frequency; purple ring indicates high centrality. **(A)** Co-citation network of references. Node size reflects citation frequency, while the color gradient from cool to warm indicates the publication recency, with cool tones for older and warm tones for recent studies. Purple circles highlight nodes with high centrality and importance. Lines between nodes represent co-citation links, and their thickness indicates the strength of these links. **(B)** Citation burst detection showing periods of intense academic interest for top 25 references. **(B)** Citation burst detection of the top 25 references, highlighting periods when these articles received significant academic attention.

The top ten cited references are listed in Table 4, with Figure 9A showing the co-citation network and Figure 9B displaying the reference burst graph. There are five references with a centrality greater than 0.1.

**Table 4:** The top 10 cited references.

Counts	Centrality	Year	Co-cited references
134	0.72	2016	Cunningham AL, 2016, New Engl J Med, V375, P1019, DOI: 10.1056/NEJMoa1603800
123	0.08	2018	Dooling KL, 2018, MMWR-Morbid Mortal W, V67, P103, DOI: 10.15585/mmwr.mm6703a5
109	0.76	2015	Lal H, 2015, New Engl J Med, V372, P2087, DOI: 10.1056/NEJMoa1501184
66	0.99	2015	Morrison VA, 2015, Clin Infect Dis, V60, P900, DOI: 10.1093/cid/ciu918
58	0.22	2005	Oxman MN, 2005, New Engl J Med, V352, P2271, DOI: 10.1056/NEJMoa051016
57	0.08	2012	Schmader KE, 2012, Clin Infect Dis, V54, P922, DOI: 10.1093/cid/cir970
51	0.14	2018	Cunningham AL, 2018, J Infect Dis, V217, P1750, DOI: 10.1093/infdis/jiy095
49	0.03	2019	Bastidas A, 2019, JAMA-J Am Med Assoc, V322, P123, DOI: 10.1001/jama.2019.9053
48	0.08	2016	Tseng HF, 2016, J Infect Dis, V213, P1872, DOI: 10.1093/infdis/jiw047
46	0.06	2014	Kawai K, 2014, BMJ Open, V4, P0, DOI: 10.1136/bmjopen-2014-004833

Oxman et al. (14) was the first to publish a study on the injection of the Oka/Merck VZV live attenuated vaccine for preventing herpes zoster. The median follow-up time was 3.12 years, and the results indicated that the vaccine could reduce the burden of herpes zoster by 61.1%, the incidence of postherpetic neuralgia by 66.5%, and the overall incidence of herpes zoster by 51.3%. The citation burst shows that this article had a significant impact between 2005 and 2010.

Lal et al. (15) from GSK demonstrated that a subunit vaccine containing varicella-zoster virus glycoprotein E and AS01 significantly reduced the risk of herpes zoster in adults aged 50 years and older. The vaccine efficacy for individuals aged 70 and above was similar to that of other age groups, with overall efficacy ranging from 96.6 to 97.9%.

The top reference is a study by Cunningham et al. (16) from GSK, which found that administering two doses of the vaccine with a two-month interval resulted in an efficacy of 89.8% against herpes zoster. Efficacy was comparable in subjects aged 70–79 years (90.0%) and those aged 80 years and older (89.1%), confirming that HZ/su can reduce the risk of herpes zoster and postherpetic neuralgia in older adults.

Cunningham et al. (17) also showed that two doses of Shingrix induced strong humoral and cellular immune responses across all age groups, particularly in those aged 70 and older, with most Shingrix recipients maintaining a significantly elevated immune response three years post-vaccination. Boutry et al. (18) reported that as of 2022, the clinical benefits of RZV for older adults persist for at least seven years post-vaccination. Studies by Anderson et al. (19) and Vink et al. (20), highlighted the significant protective efficacy of Shingrix in immunocompromised populations, solid tumor patients, and transplant recipients.

### 3.6 Visualization of disciplines

**Figure 10:**
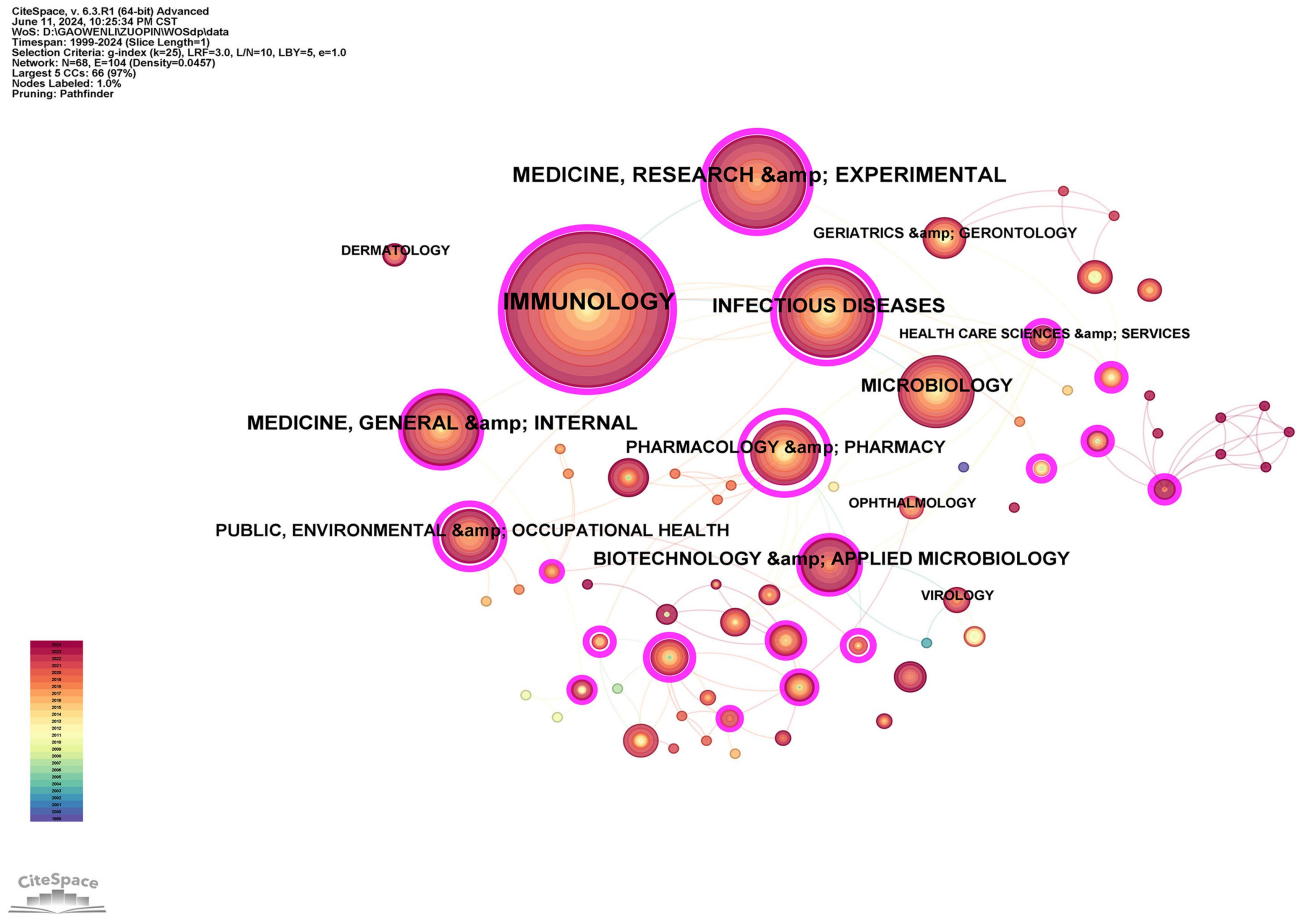
Visualization of disciplinary distribution in herpes zoster vaccine research. Node size indicates volume; edge thickness indicates cross-disciplinary linkage.

Figure 10 visualizes the disciplinary distribution of research related to herpes zoster vaccine, with parameters set to K = 25, resulting in 68 nodes (*N* = 68), 104 edges (E = 104), and a network density of 0.0457. In this co-occurrence network, each node represents a discipline, where node size corresponds to the number of publications in that discipline, and the thickness of connecting lines reflects the strength of relationships between disciplines. The color coding differentiates the various subject areas, allowing clear visualization of their interconnections.

The analysis reveals that this research field mainly involves disciplines such as dermatology, immunology, microbiology, virology, ophthalmology, infectious diseases, geriatrics and gerontology, health care sciences, pharmacology and pharmacy, as well as public, environmental, and occupational health. These disciplines correspond closely to the medical characteristics of shingles, including its age of onset, etiology, clinical symptoms, and preventive measures.

Table 5 lists the top 10 disciplines by publication count and centrality, highlighting immunology, medicine (research and experimental), infectious diseases, and pharmacology as the most prominent fields driving research in this area. Centrality values indicate the importance of each discipline within the network, reflecting their influence and connectivity to other fields.

**Table 5:** Top 10 disciplines.

Rank	Count	Centrality	Year	Discipline
1	294	0.59	2003	Immunology
2	136	0.46	2003	Medicine, Research & Experimental
3	120	0.62	2003	Infectious Disease
4	91	0.16	2005	Medicine, General & Internal
5	80	0.00	1999	Microbiology
6	65	0.16	2003	Biotechnology & Applied Microbiology
7	61	1.28	2005	Pharmacology & Pharmacy
8	47	0.73	2007	Public, Environmental & Occupational Health
9	33	0.05	2006	Geriatrics & Gerontology
10	23	0.49	2008	Health Care Sciences & Services
11	23	0.05	2003	Virology

### 3.7 Keywords co-occurrence and burst analysis

Research gaps were identified by analyzing keyword clusters and burst terms with low representation despite high clinical relevance—such as long-term safety, immune durability, and population-specific vaccination barriers. Thematic evolution also reveals a shift from early clinical evaluation to behavioral and public health issues, supporting predictions of future research focus.

**Figure 11:**
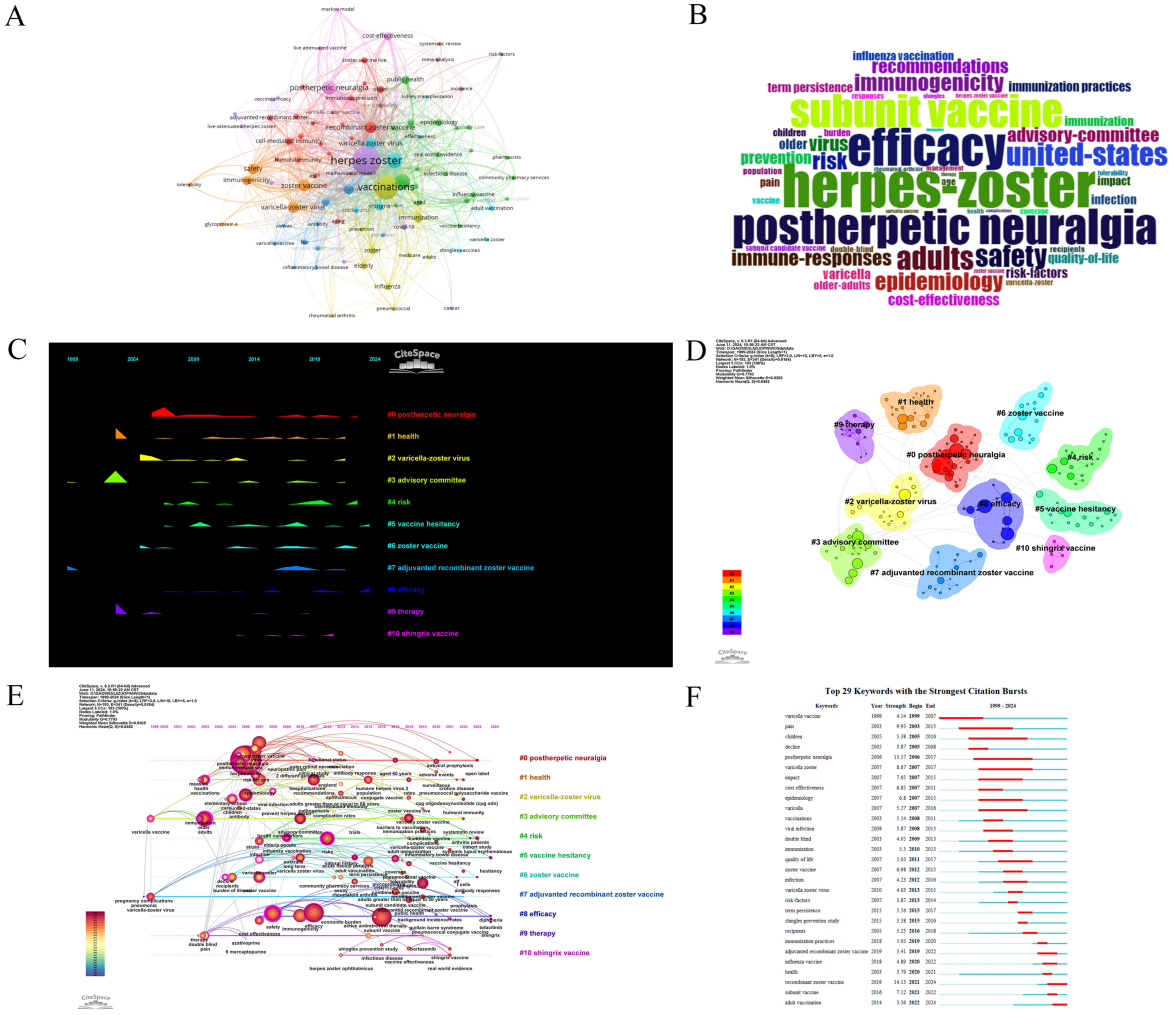
Keyword association diagrams: **(A)** co-occurrence network, **(B)** word cloud, **(C)** keyword peak map, **(D)** keyword clustering diagram, **(E)** keyword timeline, and **(F)** keyword burst diagram.

After merging keywords with similar meanings but different expressions (e.g., “herpes-zoster” and “herpes zoster”), we ultimately identified 1,719 keywords (Figures 11A,B), with 229 keywords occurring more than five times. The most frequently used keywords included “herpes zoster,” “vaccinations,” and “postherpetic neuralgia,” all of which are closely related to our research topic. Additionally, we observed high usage frequencies for terms like “efficacy,” “subunit vaccine,” and “safety,” indicating that the development and application of herpes zoster vaccines must assess both clinical effectiveness and safety.

The mountain plot in Figure 11C illustrates that since 2005, researchers have consistently increased their focus on postherpetic neuralgia, while attention to the risks of herpes zoster vaccines notably rose around 2019. In 2023–2024, the public exhibited hesitance about receiving the herpes zoster vaccine and maintained high concern regarding its effectiveness.

Our keyword clustering analysis revealed ten key clusters (see Figure 11D). Most clusters are related to themes such as postherpetic neuralgia, zoster, Shingrix, varicella-zoster virus, health, treatment, efficacy, advisory committees, vaccine hesitancy, and risks, with a primary focus on adjuvanted recombinant herpes zoster vaccines.

Figures 11E,F illustrate the keyword timeline and keyword emergence graphs, indicating a gradual increase in attention to varicella-zoster virus and pregnancy comorbidities since 1999. By 2003, there was a significant rise in focus on topics such as adults, the elderly, vaccines, pain, and immune responses. In 2005, researchers began to address disease burden, and in 2006, interest grew in topics like shingles, postherpetic neuralgia, the United States, immune responses, and acyclovir. In 2007, shingles vaccines, risk factors, cost-effectiveness, prevention, quality of life, transmission, and varicella emerged as hot topics. By 2008, safety and viral infections became focal issues, with prevention of shingles recognized as a key research area in 2009.

These keyword evolution patterns reveal three major thematic shifts in herpes zoster vaccine research over the past two decades: (1) From early focus on disease burden and antiviral treatments (1999–2005), (2) to expansion in vaccine development and clinical efficacy studies (2006–2015), and (3) to recent focus on vaccine hesitancy, immunocompromised populations, and real-world data (2016–2024). This timeline reflects a transition from foundational clinical trials to public health application.

In 2010, immunogenicity and advisory committees gained prominence, with a focus on efficacy and recommendations in 2011, and broad concern about risk issues in 2012. In 2013, interest shifted to cell-mediated immunity and ocular herpes zoster; in 2014, the relationship between rheumatoid arthritis and herpes zoster came under scrutiny (21). In 2016, subunit vaccines became a research focus, and in 2017, studies on CD4 + T cells, vaccine coverage, glycoprotein E, and populations aged 50 and older emerged. By 2018, immunization practices and public health were major focal points, followed by a concentration on adjuvanted recombinant herpes zoster vaccines in 2019. In 2020, the focus shifted to adverse events, while 2021 saw emphasis on vaccine hesitancy, real-world data, and reactivation. In 2022, attention was given to humoral immunity and T cells, in 2023 to cohort studies, open-label trials, and patients with arthritis (22), and in 2024 to vaccine hesitancy and the Shingrix vaccine as key topics.

### 3.8 Expanded analysis on thematic hotspots and underlying controversies

Although “efficacy” and “safety” emerged as the most frequently co-occurring keywords (Figures 11A–F), they represent more than just frequency—they highlight ongoing controversies in vaccine science. For instance, while both Zostavax and Shingrix show high efficacy in clinical trials, real-world studies reveal a divergence in long-term immune protection, especially among immunocompromised populations (23). There is ongoing debate regarding the sufficiency of safety data, particularly in older adults and those with comorbidities (23).

Furthermore, the keyword “vaccine hesitancy” has shown a strong burst in 2023–2024. This rise coincides with increased public skepticism about pharmaceutical transparency and government recommendations during and post-COVID-19 (24). Studies have indicated that pandemic-era communication gaps, concerns over rapid vaccine development timelines, and inconsistent messaging contributed to broader distrust in vaccination campaigns (24, 25). These sociopolitical factors must be considered alongside biomedical evidence to understand the full context of hesitancy.

In future research, multidisciplinary analysis combining clinical data, behavioral science, and public health communication is crucial to better understand vaccine acceptance patterns and address hesitancy effectively (26).

## 4 Discussion

This study presents the first systematic bibliometric analysis of herpes zoster (HZ) vaccine research over the past 25 years. By employing a multidimensional approach with tools including CiteSpace, VOSviewer, R-bibliometrix, SCImago, and online bibliometric platforms, we comprehensively analyzed global research output across multiple domains—publication trends, geographic and institutional contributions, authorship, journal impact, disciplinary intersections, citation networks, and evolving research topics. This integrative analysis provides a panoramic view of the development trajectory of HZ vaccine research and identifies key hotspots and potential future directions.

### 4.1 Research trends and global distribution

The overall volume of HZ vaccine-related publications has steadily increased, with two distinct surges: the first following the development and release of the live attenuated vaccine Zostavax in 2006 by Oxman et al., and the second linked to the 2017 introduction of the recombinant subunit vaccine Shingrix. These inflection points reflect significant technological and clinical advancements in the field, and publication activity peaked in 2021. The enduring annual output exceeding 50 articles since 2018 underscores sustained research interest.

Geographically, the United States has emerged as the dominant contributor, both in publication quantity and international collaboration. This leadership is supported by major academic institutions and pharmaceutical enterprises, notably GlaxoSmithKline (GSK) in the United Kingdom. High-impact collaborations, particularly with Finland, further emphasize the importance of global research networks in driving innovation.

### 4.2 Interdisciplinary and institutional contributions

HZ vaccine research is inherently interdisciplinary. While the core contributions are concentrated in immunology, infectious diseases, and clinical medicine, related disciplines such as pharmacology, epidemiology, and public health play essential roles in translating research into real-world applications. This interdisciplinary integration is vital for advancing vaccine development, evaluating health economics, and guiding implementation strategies.

Analysis of institutional productivity revealed that seven of the top 11 institutions are based in the U.S., and that authors such as Levin et al. have played pivotal roles in foundational vaccine trials. The citation analysis shows that journals like *Vaccine*, *Human Vaccines & Immunotherapeutics*, and *The Journal of Infectious Diseases* serve as key publishing venues, while *The New England Journal of Medicine* remains the most frequently cited source, indicating its influential role in disseminating critical clinical findings.

According to the Journal Citation Reports (JCR) from Thomson Reuters, the Impact Factor (IF) (27) is widely recognized as an important indicator of journal quality and influence. Among the journals involved in this field, *Vaccine* holds the highest volume of publications, followed by *Human Vaccines & Immunotherapeutics* and *The Journal of Infectious Diseases*. In the citation analysis, *The New England Journal of Medicine* ranks first with 556 citations and boasts an exceptionally high impact factor of 158.5, placing it in the Q1 quartile. The inclusion of impact factor data provides an objective measure to evaluate the academic standing of these journals within the HZ vaccine research field, offering researchers reliable guidance for literature selection.

While institutional and national output metrics highlight dominant contributors, it is essential to explore the underlying drivers of this research productivity. For instance, GlaxoSmithKline’s prominence is likely not coincidental, as it reflects the company’s strategic investment in the development and commercialization of Shingrix, which has shaped global vaccination efforts (28, 29). Similarly, the United States’ leadership may not only stem from research volume but also from the robust infrastructure supporting translational science and policy implementation. However, prior research indicates that dominance in publication quantity does not always reflect higher methodological quality (28).

For instance, GSK’s high publication output is closely tied to its development and global promotion of Shingrix, which has driven both clinical trials and affiliated scientific publications.

Additionally, the relatively sparse collaboration networks observed in bibliometric maps may suggest existing barriers to international or interdisciplinary cooperation. These could include institutional silos, resource inequalities, or competitive research funding mechanisms that disincentivize open collaboration (29). Encouraging broader, cross-border academic exchange—especially involving underrepresented regions—may be critical to advancing equitable progress in HZ vaccine research.

### 4.3 Hotspots and thematic evolution

Thematic mapping and keyword co-occurrence analyses highlight the field’s evolution from early clinical trials to the exploration of broader vaccination strategies. Recent research focuses on vaccine effectiveness in special populations—older adults, immunocompromised patients, and individuals with comorbidities such as diabetes (30), cardiovascular disease (31), cerebrovascular disease (32), dementia (33, 34), inflammatory bowel disease (IBD) (35, 36), and systemic lupus erythematosus (SLE) (37, 38). These findings reinforce the expanding recognition of herpes zoster as a serious public health concern across diverse clinical settings.

Future researchers should prioritize longitudinal, multidisciplinary studies that integrate immunological, behavioral, and socio-political dimensions to better address emerging challenges such as vaccine hesitancy and access disparities.

Combination vaccine strategies and novel adjuvants represent current research frontiers. Studies demonstrate that both live attenuated (39) and recombinant vaccines (22) are safe and immunogenic when co-administered with pneumococcal or influenza vaccines (40). Meanwhile, the recombinant zoster vaccine (RZV), containing glycoprotein E with the AS01B adjuvant, has shown high durability of protection (>10 years). However, the limited availability of QS-21, a key adjuvant component, has prompted investigations into alternative formulations, such as CpG oligonucleotides and lipid nanoparticle systems.

Furthermore, combination vaccination strategies have gained increasing attention Clinical studies have confirmed that both ZVL and RZV can be co-administered with commonly used adult vaccines. Zostavax, when administered with the pneumococcal polysaccharide vaccine (PPV23) (41) or quadrivalent inactivated influenza vaccine (IIV4) (42), demonstrates acceptable safety profiles. Similarly, RZV has been shown to maintain high immunogenicity and safety when given alongside the 13-valent pneumococcal conjugate vaccine (PCV13) (43) or PPV23 (44, 45). Comparative analyses across countries such as Germany (46), Italy (47), and Australia (48) consistently indicate that recombinant subunit vaccines offer greater protection and longer immune persistence than live attenuated vaccines (49). While ZVL’s efficacy tends to wane 3–7 years post-vaccination, it can be partially restored with revaccination (50). In contrast, RZV provides durable protection exceeding 10 years (51, 52). The AS01B adjuvant in Shingrix plays a central role in its efficacy; however, the limited availability of its critical component QS-21 has prompted exploration into alternative adjuvant platforms such as CpG oligonucleotides and ionizable lipid nanoparticles (53–55), which are currently under investigation for future vaccine formulations (56).

### 4.4 Translational challenges and public health gaps

Despite robust evidence supporting the efficacy and safety of herpes zoster vaccines—particularly the recombinant zoster vaccine (RZV)—real-world vaccination rates remain low. Several multifactorial barriers contribute to this gap. On one hand, public awareness of the vaccine remains inadequate (57), and willingness to be vaccinated is influenced by demographic, socioeconomic, and geographic factors (58). On the other hand, there is considerable variability in healthcare providers’ knowledge (59) and prescribing behaviors, indicating that vaccination recommendations from physicians need to be strengthened (60).

Additionally, logistical and systemic obstacles—such as limitations in national reimbursement policies and the high costs associated with cold-chain storage—further hinder vaccine accessibility (61). These real-world challenges persist even in countries where vaccines are widely available and recommended (60).

Studies from various countries also illustrate how health system design and public education affect HZ vaccine uptake. For example, a national survey in South Korea found that physician recommendation was a strong predictor of vaccine uptake, despite low awareness levels among patients. In Mexico, structural limitations—such as inadequate cold-chain infrastructure and budget constraints—continue to obstruct adult immunization programs.

These findings emphasize the urgent need for coordinated strategies that span biomedical innovation, healthcare provider education, and systems-level policy reform. Bibliometric analysis, in this context, offers not only a retrospective view of scientific activity but also valuable insight for guiding future implementation strategies tailored to real-world challenges.

### 4.5 Emerging scientific and sociopolitical challenges

While the high efficacy and safety profiles of HZ vaccines, particularly Shingrix, are well-supported by clinical trials, concerns remain regarding the completeness and transparency of long-term safety data—especially in frail elderly and immunocompromised populations. For instance, real-world studies have raised questions about the duration of immune protection, and variability in T-cell–mediated immunity across age groups. Furthermore, mechanisms underlying long-lasting immunity from recombinant subunit vaccines, such as the role of CD4 + T cells and persistent antigen presentation, warrant further elucidation.

The rising vaccine hesitancy observed in 2023–2024 also reflects a broader post-pandemic phenomenon. COVID-19 reshaped public trust in health institutions and vaccines, amplifying skepticism due to inconsistent messaging, politicization of science, and increased exposure to misinformation. These factors likely spill over into perceptions of other adult vaccines, including HZ vaccines (62, 63). Future research should integrate behavioral science, epidemiological modeling, and digital media analysis to better understand and address these sociopolitical dimensions of vaccine uptake.

### 4.6 Global perspective and health inequities

As a bibliometric study, this discussion lacks a sufficiently comprehensive global perspective. The current interpretation overly emphasizes research outputs from major countries, particularly the United States and Western Europe, while overlooking several critical dimensions. First, it is necessary to examine whether publication patterns genuinely reflect scientific progress or are influenced by pharmaceutical industry interests. Previous studies have demonstrated that industry sponsorship can significantly affect research outcomes and priorities (64, 65). Second, regions with high herpes zoster incidence but limited literature representation, such as Southeast Asia and Africa, may have different research focuses, highlighting broader issues of global health inequity (66). Third, the inclusion criterion restricted to English-language publications introduces potential language bias, limiting the comprehensiveness of findings regarding non-English-speaking countries (67, 68). To achieve a more thorough and equitable global perspective, these factors should be carefully considered in bibliometric analyses, situating the findings within the wider context of global health disparities.

## 5 Limitations

Several limitations must be acknowledged. First, this study relied exclusively on the Web of Science Core Collection, potentially omitting relevant research indexed in other databases such as Scopus, PubMed, or Embase. Second, non-English-language publications were excluded, which may underrepresent research outputs from non-English-speaking countries. Third, data from 2024 are incomplete due to the real-time nature of indexing. Fourth, while bibliometric tools allow for efficient trend identification, they do not assess study quality, clinical relevance, or outcome rigor as traditional or AI-assisted systematic reviews do. However, our bibliometric analysis employed rigorous search strategies and combined automated and manual quality controls to ensure data reliability. This approach provides a unique macro-level perspective on research trends, collaboration patterns, and knowledge gaps, thereby complementing other review methods and supporting informed research and policy decisions. Lastly, name disambiguation challenges could introduce minor inaccuracies in author-level metrics, although these are unlikely to affect overall trends or conclusions.

## 6 Future directions and research implications

Despite substantial progress in HZ vaccine research, several areas merit further investigation:

Long-term Immunity: Clarifying the immunological mechanisms that confer sustained protection in elderly and immunocompromised populations is essential for optimizing vaccine schedules and booster strategies.

Combination and Booster Strategies: Future research should evaluate co-administration protocols and long-term outcomes through large, multicenter randomized controlled trials.

Technological Innovation: Advances in adjuvant development (e.g., CpG ODNs, ionizable lipid nanoparticles) and recombinant platforms could lead to next-generation vaccines with enhanced durability and fewer logistical constraints.

Population-Specific Approaches: Tailored vaccine designs targeting high-risk subgroups, including those with autoimmune or metabolic conditions, should be prioritized.

Global Equity and Data Sharing: Enhanced international collaboration and real-world data integration are critical for addressing disparities in vaccine access, especially in low- and middle-income countries.

Health System Interventions: Targeted educational initiatives for both healthcare professionals and the public, combined with supportive reimbursement policies and infrastructure investment, are needed to improve vaccine uptake.

This study provides researchers and policymakers with an evidence-based framework to identify and close knowledge gaps, ultimately informing global efforts to strengthen HZ prevention through effective vaccination.

## 7 Conclusion

This bibliometric review offers a comprehensive analysis of the global herpes zoster vaccine research landscape over the past two and a half decades. The United States leads in research output and collaboration, with GSK and other institutions contributing significantly to vaccine development and implementation science. The transition from live attenuated to recombinant subunit vaccines has reshaped the field, but challenges in vaccine accessibility and uptake persist. Our findings serve as a valuable resource for guiding future research agendas, clinical strategies, and policy initiatives aimed at maximizing the public health impact of HZ vaccination.

## Data Availability

The raw data supporting the conclusions of this article will be made available by the authors, without undue reservation.

## Footnotes

^1^ https://bibliometric.com
